# An Overview of the Treatment Strategies of Extremities Ischemia in the Intensive Care Unit

**DOI:** 10.7759/cureus.33454

**Published:** 2023-01-06

**Authors:** Tuğba T Onur, Asiye A Demirel

**Affiliations:** 1 Department of Anesthesiology and Reanimation, University of Health Sciences Bursa Yüksek İhtisas Training and Research Hospital, Bursa, TUR

**Keywords:** peripheral vascular disease, amputation, femoral nerve block, intensive care unit, peripheral block

## Abstract

Objectives: To investigate the effect of the peripheral block on peripheral ischemia on the extremities of patients in the intensive care unit (ICU).

Materials and Methods: Sixty-two patients with ischemic peripheral vascular disease were divided retrospectively into two groups; Group 1 (patients who underwent USG-guided infraclavicular or femoral block, n=20) and Group 2 (patients who did not experience any block, n=42). The demographic characteristics of the patients, the diagnosis of hospitalization, the day when the circulatory disorder developed, laboratory tests at the time of diagnosis, other medical treatments applied, presence of inotropic support, the response of ischemia on extremities, amputation, duration of hospital stay, discharge and mortality were compared.

Results: The most common reason for hospitalization was cerebrovascular disease. There was no statistical difference between the groups regarding age, gender, height, body weight, and diagnosis. There was no statistical difference between the groups regarding hematocrit, lactate, creatinine, and albumin values, the day when the peripheral ischemia developed in extremities, inotropic and prednisolone use, presence of cannulation, length of hospital stays, and mortality. The number of patients discharged from the intensive care unit in Group 1 was significantly higher than in Group 2 (p=0.048). Amputation was performed on one patient in Group 1 and two patients in Group 2. There was a decrease in peripheral ischemia in 14 (70%) of the patients in Group 1 and 25 (59.5%) of the patients in Group 2.

Conclusions: Targeted peripheral block techniques for peripheral circulatory disorders for selected ICU patients in conjunction with preventive and medical treatments may decrease peripheral ischemia in extremities and increase ICU discharge.

## Introduction

Invasive arterial cannulations, which are frequently used in the monitoring and treatment of hemodynamic disorders in ICU patients, using vasopressor drugs, coagulopathies, peripheral vascular and connective tissue diseases, hypovolemic shock, septic embolisms, medicines such as steroids, accompanying autonomic dysfunctions and extremity distal ends are risk factors for the development of ischemic complications [1]. Among the treatments recommended for peripheral circulatory disorders are the termination of cannulation, elevation, warming, anticoagulants, and/or vasodilator agents. Avoidance of unnecessary vasopressor drug use, peripheral block application, and surgical techniques, including amputation in more severe cases, are also treatment options [2]. As primary prophylaxis for peripheral vascular ischemia, anticoagulant therapy is often administered in ICUs [3]. There is evidence that elevating the extremities is beneficial in preventing peripheral ischemia in extremities associated with edema and compartment syndrome. In addition to medical and preventive treatment, it has recently been reported that sympathetic ultrasound-guided peripheral blocks can increase tissue perfusion, decrease ischemic pain, and resolve vasodilation and reflex vasospasm [3]. Ischemic extremity treatment aims to reduce ischemia pain, increase blood flow, and prevent limb loss associated with ischemia [4]. The most severe outcome of peripheral circulatory disorders is amputation, likely caused primarily by vasospastic factors. Vasospasm caused by a patient's underlying critical illness and vasoactive agents should usually resolve as the patient's general condition improves and vasoactive agents are withdrawn [5].

In this study, we aimed to retrospectively examine the characteristics of patients with peripheral ischemia in extremities during hospitalization in the ICU, their treatment approaches, and the results of their treatment.

## Materials and methods

A retrospective evaluation of patients hospitalized in the ICU at the University of Health Sciences Bursa Yüksek İhtisas Training and Research Hospital, Bursa, Turkey, between January and September 2022 was conducted with the approval of the Ethics Board of the University of Health Sciences Bursa Yüksek İhtisas Training and Research Hospital under decision number 2011-KAEK-25 2022 09-07. Malignancy or trauma-related circulatory disorders, patients with peripheral arterial disease and circulatory disorders before ICU admission were excluded from the study. Demographic characteristics of the patients (age, gender, height, weight), primary diagnoses, medications, presence of invasive cannulation in the extremity with ischemia, use of vasoactive agents, use of anticoagulants, use of steroids, laboratory (hematocrit, leukocyte, creatinine, albumin ) values at the time of diagnosis, the day of detection of peripheral ischemia in extremities, the treatment approaches (warming, elevation, peripheral block ), consultations, surgical intervention or amputation, if any, and mortality data were evaluated. Patients were divided into two groups: Group 1 (n=20; 8 patients with continuous infusion and 12 patients with bolus dose), who underwent ultrasound-guided peripheral block, and Group 2 (n=42), who did not experience peripheral block for peripheral vascular ischemia. The peripheral block cases included patients who had undergone infraclavicular and femoral block methods.

Peripheral vascular disease

The diagnosis of ischemic peripheral disease was made by the presence of cyanosis, especially in the extremities, in the daily physical examination of the patient. The extremity and the extent of cyanosis of the patients with cyanosis on inspection were recorded on the follow-up papers. In our intensive care unit, heating and elevation of the extremity with circulatory disorders are performed for every patient with cyanosis. Extremity examinations record whether there is progression or regression in circulatory disorder during intensive care stay. Doppler USG has been performed on all patients with extremity ischemia to rule out peripheral thromboembolism.

Peripheral block method

A perifix® 401 epidural catheter set (Braun Medical, Germany) was used to apply the peripheral nerve block. The location was determined via the linear probe of the ultrasound device (GE Healthcare, Wauwatosa, Wisconsin, USA) and depending on the location of the circulatory disorder. A catheter was inserted around the nerve to apply the block. A solution prepared with 120 mL saline and 40 mL 0.125% bupivacaine (Marcaine®) through the catheter was administered as a continuous infusion at a rate of 5 ml/hour or a bolus of 15 mL 0.125% bupivacaine (Marcaine®) + 5 mL 2% lidocaine (Aritmal®).

Management of hypotension

In the ICU, hypotension was defined as a systolic blood pressure less than 90 mmHg and a mean arterial pressure less than 60-65 mmHg, or a systolic blood pressure value that fell below the basal systolic blood pressure of 40 mmHg in hypertensive patients. Crystalloids or colloids were used for fluid resuscitation, with a central venous pressure goal of 8-12 mmHg. A vasopressor therapy, deemed appropriate by the following intensive care specialist, was initiated if the patients were still hypotensive after the central venous pressure reached the target value.

Statistical analysis

Descriptive data were presented as numbers and percentages, and measurement data were presented as mean ± standard deviation. Shapiro-Wilk test and histogram graphs were used to examine the assumption of normal distribution of the measurements. Chi-square and Fisher's Exact were used to compare categorical data appropriate for the test usage area. The Mann-Whitney U test was used in independent groups to compare non-normally distributed measurements. P<0.05 was accepted for statistical significance. All analyzes were performed with SPSS version 20.0 (IBM Corp., Armonk, NY).

## Results

Sixty-two patients who developed peripheral ischemia in extremities during ICU hospitalization were evaluated. Patients who underwent local anesthetic with ultrasound-guided peripheral nerve block were selected as Group 1 (n=20), and those who did not use any peripheral nerve block method were designated as Group 2 (n=42). In Group 1, the infraclavicular block was applied to eight patients, and the femoral block was used on 12 patients. There was no statistical difference between the groups regarding patient characteristics and diagnosis of ICU hospitalization. The general characteristics of the patients are given in Table 1.

**Table 1:** General features of the patients (Mean ± Std, n%) cm: Centimeters, kg: Kilograms, CVD: Cerebrovascular disease Other: Gastrointestinal bleeding, Acute renal failure.

		Group 1	Group 2	P
Gender	Female n (%)	8 (40)	19 (45.2)	0.909
	Male n (%)	12 (60)	23 (54.8)	
Age (year)		72.00±8.68	73.57±11.00	0.845
Height (cm)		166.05±5.43	167.02±5.53	0.565
Weight (kg)		73.15±15.28	77.52±15.92	0.553
CVD	n (%)	7 (35)	11 (26.2)	0.678
Respiratory fairly	n (%)	3 (15)	12 (28.6)	0.243
Sepsis	n (%)	3 (15)	8 (19)	0.697
Other	n (%)	7 (35)	11 (26.2)	0.678

There was no statistical difference between the groups in terms of hematocrit, lactate, creatinine, and albumin values recorded on the day of diagnosis of the peripheral ischemia in extremities, day of onset circulatory disorder, ICU length, and mortality rates (Table 2). There were significantly more patients transferred to the service in Group 1 as compared to Group 2 (p=0.048).

**Table 2:** Analyzing perioperative data according to study groups (Mean ± Std, n%) *p<0.05 Fisher exact test

	Group 1	Group 2	P
Hematocrit (%)	26.85±2.45	26.94±3.08	0.964
Creatinine (mg/dl)	1.24 ± 1.00	1.38 ± 0.94	0.550
Albumin (g/l)	22.63±3.24	26.58±2.71	0.208
Lactate (mmol/l)	4.92 ± 2.00	5.63 ± 2.15	0.271
The beginning day of peripheral ischemia on ICU (day)	10.50 ± 7.04	12.54 ± 8.76	0.473
ICU hospitalization (day)	41.30±23.58	41.40±22.80	0.934
Patients transferred to the service n (%)	8 (40)	6 (14.3)	0.048*
Patients transferred to palliative n (%)	6 (30)	15 (35.7)	0.657
Mortality n (%)	6 (30)	21 (50)	0.226

There was no statistical difference between the groups in terms of vasopressor use, the presence of intraarterial cannulation, and methylprednisolone use (Table 3).

**Table 3:** Comparison of inotrope requirements, methylprednisolone use, and intraarterial cannulation presence between the groups (n%)

	Group 1	Group 2	p
Patient with dopamine+dobutamine infusion n(%)	12 (60)	23 (54.8)	0.861
Patient with norepinephrin+epinephrin infusion n(%)	4 (20)	12 (28.6)	0.861
Patient with methylprednisolone therapy n (%)	5 (25)	11 (26.2)	0.629
Patient with intraarterial cannulation when ischemic peripheric disorder develops n (%)	7 (35)	9 (21.4)	0.254

All patients who developed peripheral ischemia were treated with warming, elevation, glyceryl trinitrate infusion, and low molecular weight heparin (LMWH). Two patients in Group 1 and six in Group 2 were treated with dextran, low molecular weight. One patient in Group 1 and two in Group 2 underwent amputations following surgery recommendations. There was a decline in peripheral cyanosis in 14 patients (70%) in Group 1 and 25 patients (59.5%) in Group 2.

## Discussion

According to our study evaluating our approaches to ischemia of extremities in our ICU, the number of patients transferred to the service was higher in the patient group (Group 1) who underwent peripheral nerve blockage. Additionally, healing from ischemia of extremities was observed more frequently in Group 1. In terms of demographic data, hospitalization diagnoses, laboratory tests (hematocrit, lactate, creatinine, and albumin), intraarterial cannulation status, the need for vasopressor support, methylprednisolone use, ICU hospitalization, and mortality rates, both groups had similar characteristics.

Treating patients with extremity ischemia is first performed by removing the occlusive tissue from the extremity artery, warming the extremity, adding anticoagulant and thrombolytic treatments in case of continued ischemia, or using interventional techniques (stellate ganglion or brachial plexus sympathetic blockade). Brachial plexus or stellate ganglion block reduce ischemia by causing vasodilation due to sympathetic blockade, providing circulation in the distal extremity, reducing ischemia pain, and eliminating ischemia reflex vasospasm [6,7]. Continual infusion and medical treatment effectively treat extremity ischemia caused by accidental intraarterial injection with a plexus block [8]. A femoral block performed on a right femoral circulation disorder patient prevented the patient from below-knee amputation [9]. Sepsis and hypercoagulation triggered by cytokine storms may lead to endothelial damage that may lead to increased rates of circulatory disorders. A femoral block prevents vasoconstriction, peripheral vascular resistance decreases in response to vasodilation, and new thrombus formation is prevented. Consequently, distal extremity circulation is improved, and ischemia is reduced [9]. Our study revealed similar results to other studies in that peripheral block patients' circulatory disorders decreased more than those in the other group. In addition, they were more likely to be discharged from the ICU. The lack of difference between the groups regarding amputation and mortality suggests that block application does not contribute to survival.

Sepsis is accompanied by a hyperinflammation syndrome associated with excessive cytokine secretion. Inotropes are used in the non-fluid phase of sepsis. Here, adrenaline or noradrenaline is recommended as the first drug, but if it is not available, dopamine can be used. There is no clear information about the benefit of hydrocortisone use in patients whose shock symptoms do not improve despite fluid and vasopressor therapy. Hydrocortisone can be administered in patients unresponsive to fluids and vasopressors [10]. Dopamine has dose-dependent inotropic effects. It is emphasized that even at low doses, peripheral digit infarcts may occur due to vasoconstriction [11,12]. A study reported an increased risk of arrhythmia and digital ischemia in patients using dopamine after cardiac surgery compared to those using norepinephrine [13]. In another study, vasopressin was used in patients with septic shock. While it caused more digital ischemia than norepinephrine, mortality rates were similar [14]. The presence of arterial lines was also associated with finger ischemia in patients hospitalized in intensive care. Anticoagulation or antiplatelet therapy has been indicated as appropriate therapy. Although progression to amputation is rare, it has high mortality in patients with finger ischemia in intensive care, especially in the presence of cancer. Non-ICU patients hospitalized with finger ischemia require frequent finger amputations, possibly because of more common connective tissue disorders [15]. Several reports of upper and lower extremity digital ischemia have been published in patients receiving vasopressor agents. Pre-existing vascular disease, simultaneous use of multiple inotropic agents, and prolonged periods of hypotension are risk factors for digitalis circulatory disorder [16]. It is prudent to minimize the dosage and duration of vasopressor use as much as possible. The use of anticoagulation or antiplatelet therapy is not well established, although these patients often take these drugs for other indications. In cases of arterial thrombosis, these treatments are warranted; however, the benefit of adding these agents in severe cases of vasospasm is questionable. In our research, vasopressor support in 16 patients in Group 1 and 35 patients in Group 2 suggests that peripheral circulatory disorders are associated with vasopressors. In general, the combination of inotropic support and the accompanying infections of the patients prevented us from commenting on which vasopressor was riskier in terms of peripheral circulation. In addition, we observed that the groups were similar in the presence of cannulation, methylprednisolone use, and prophylactic LMWH treatment used in all patients.

The determination of an extremity at risk is critical to ascertain the healing potential and the level of amputation to be applied if necessary. In our study, peripheral ischemia in extremities increased in both groups despite medical treatment and peripheral block approaches, and patients who underwent amputation were observed. Amputation was performed on one patient in Group 1 and two patients in Group 2 with the joint decision of the orthopedics and cardiovascular surgery team. In a study, amputations were associated with deficient serum albumin levels and increased mortality in unstable cardiac patients [16]. In another study, risk factors for amputation were reported as being over 60 years old, having ankle-brachial index (ABI) <0.80, having a monomicrobial infection, having white blood cell count >15x109, having ESR>100mm/hour, having hemoglobin ≤10.0g/dL and creatinine≥150µmol/L as gangrenous wound type [17]. Since the number of amputations was deficient in our study, we could not examine the relationship between patient features and laboratory tests.

Study limitations

The most important limitation of the study is the small number of patients. Insufficient studies in the literature regarding peripheral circulatory disorders developing in intensive care units led to difficulties in terms of definition in the methodology.

## Conclusions

Circulatory disorders in the peripheral extremities, which can be triggered by many reasons such as drugs used in intensive care units, sepsis, and immobilization, are a common problem with no consensus on treatment strategies. In this study, it has been concluded that ultrasound-guided peripheral nerve blocks are associated with increased ICU discharge and healing in ischemia of extremities. Even though peripheral block methods do not reduce mortality, they can be an effective treatment option for managing peripheral ischemia of extremities. We think that in the approach to treating peripheral ischemia of extremities in intensive care units, preventive measures accompanying etiological treatment and peripheral block methods that can be applied in addition to medical treatments should be the subject of further research.

## References

[1] Valentine RJ, Modrall JG, Clagett GP (2005). Hand ischemia after radial artery cannulation. J Am Coll Surg.

[2] Brzezinski M, Luisetti T, London MJ (2009). Radial artery cannulation: a comprehensive review of recent anatomic and physiologic investigations. Anesth Analg.

[3] Sen S, Chini EN, Brown MJ (2005). Complications after unintentional intra-arterial injection of drugs: risks, outcomes, and management strategies. Mayo Clin Proc.

[4] Varu VN, Hogg ME, Kibbe MR (2010). Critical limb ischemia. J Vasc Surg.

[5] Obritsch MD, Jung R, Fish DN, MacLaren R (2004). Effects of continuous vasopressin infusion in patients with septic shock. Ann Pharmacother.

[6] Özdinç O, Evren Şahin K, Göktay A, Onay M, Tarcan E (2013). Yanlışlıkla intra-arteriyel atrakuryum enjeksiyonu yapılan pediatrik olgu (Article in Turkish). Ege Tıp Dergisi.

[7] Soberón JR, Duncan SF, Sternbergh WC (2014). Treatment of digital ischemia with liposomal bupivacaine. Case Rep Anesthesiol.

[8] Tütüncü AÇ, Kendigelen P, Altıntaş F, Güner K (2017). Use of continuous brachial plexus blockade for treatment of accidental intra-arterial injection. Journal of Emergency Medicine Case Reports.

[9] Uluç K, Ervatan Z, Gün Í, Mutlu S, Turgut N (2022). Alt Ekstremite Arter Oklüzyonunda Femoral Blok Uygulaması Yapılan COVID-19 Hastası (Article in Turkish). Turkish Journal of Intensive Care.

[10] Weiss SL, Peters MJ, Alhazzani W (2020). Surviving sepsis campaign international guidelines for the management of septic shock and sepsis-associated organ dysfunction in children. Intensive Care Med.

[11] Uzun U, Balik Y Anestezide Hasta Takibi. 2022.

[12] Träger K, DeBacker D, Radermacher P (2003). Metabolic alterations in sepsis and vasoactive drug-related metabolic effects. Curr Opin Crit Care.

[13] Hajjar LA, Vincent JL, Barbosa Gomes Galas FR (2017). Vasopressin versus norepinephrine in patients with vasoplegic shock after cardiac surgery: the VANCS randomized controlled trial. Anesthesiology.

[14] Nagendran M, Russell JA, Walley KR (2019). Vasopressin in septic shock: an individual patient data meta-analysis of randomised controlled trials. Intensive Care Med.

[15] Landry GJ, Mostul CJ, Ahn DS (2018). Causes and outcomes of finger ischemia in hospitalized patients in the intensive care unit. J Vasc Surg.

[16] Daroca-Pérez R, Carrascosa MF (2017). Digital necrosis: a potential risk of high-dose norepinephrine. Ther Adv Drug Saf.

[17] Aziz Z, Lin WK, Nather A, Huak CY (2011). Predictive factors for lower extremity amputations in diabetic foot infections. Diabet Foot Ankle.

